# Rapid Cycle Deliberate Practice in Medical Education - a Systematic Review

**DOI:** 10.7759/cureus.1180

**Published:** 2017-04-19

**Authors:** Jillian Taras, Tobias Everett

**Affiliations:** 1 Anesthesiology Resident, University of Toronto, Canada; 2 Department of Anesthesia, The Hospital for Sick Children, University of Toronto

**Keywords:** rapid cycle deliberate, systematic review, simulation, medical education

## Abstract

Rapid Cycle Deliberate Practice (RCDP) is a novel simulation-based education model that is currently attracting interest, implementation, exploration and research in medical education. In RCDP, learners rapidly cycle between deliberate practice and directed feedback within the simulation scenario until mastery is achieved. The objective of this systematic review is to examine the literature and summarize the existing knowledge on RCDP in simulation-based medical education. Fifteen resources met inclusion criteria; they were diverse and heterogeneous, such that we did not perform a quantitative synthesis or meta-analysis but rather a narrative review on RCDP.

All resources described RCDP in a similar manner. Common RCDP implementation strategies included: splitting simulation cases into segments, micro debriefing in the form of ‘pause, debrief, rewind and try again’ and providing progressively more challenging scenarios. Variable outcome measures were used by the studies including qualitative assessments, scoring tools, procedural assessment using checklists or video review, time to active skills and clinical reports. Results were limited and inconsistent. There is an absence of data on retention after RCDP teaching, on RCDP, with learners from specialties other than pediatrics, on RCDP for adult resuscitation scenarios and if RCDP teaching translates into practice change in the clinical realm. We have identified important avenues for future research on RCDP.

## Introduction and background

In the continuous evolution of education practices, a current emerging modality is rapid cycle deliberate practice (RCDP) simulation-based learning. Early research is focusing not only the efficacy of the modality but also how it compares to other types of simulation-based learning and what characteristics of RCDP are associated with the greatest effect on learning, retention, and impact on patient care.

Several systematic reviews have evaluated the effectiveness of simulation-based medical education (SBME) and variations thereof and as a community, we are satisfied that SBME in the correct context, it offers an advantage over traditional medical education modalities [1-2]. Research in this area now focuses on the manner in which SBME can be employed to greatest advantage.

Two established variations of SBME are deliberate practice (DP) and mastery learning (ML). Deliberate practice is the key to the development of expertise in many fields (e.g. sports, aviation, chess, music, academia) and importantly in clinical competence [3-6]. Mastery learning has also been shown as a successful learning model in medicine with evidence supporting every level of impact from bench to bedside [7-8]. Both approaches have been subjected to an extensive investigation of their effectiveness and data from studies have been synthesized to convince us that DP and ML are useful tools [5, 9-16]. 

According to a recent systematic review, the two most cited features of SBME that lead to effective learning are feedback and repetitive practice [3,17]. However, there is still limited empirical evidence that supports specific methods of feedback and debriefing over others [18].

Recent review articles on feedback and debriefing provide evidence supporting both post-simulation debriefing and within-simulation debriefing [18-20]. Post-simulation debriefing is most commonly used and various studies have shown that it promotes effective learning and retention in SBME [18, 20-24]. Within-simulation debriefing has been shown beneficial in improving technical skills, adherence to resuscitation guidelines and achieving mastery learning goals [18-19, 25-26]. Authors suggest that within-event feedback is effective due to the 'self-determination theory'. This means that learners receive feedback, repeat the task and see themselves improve, which promotes feelings of competence and allows learners to welcome feedback [20]. Alongside the merit of within-simulation debriefing, we also know that repeating a scenario confers learning benefit [27].

Rapid cycle deliberate practice (RCDP), coined by Hunt in 2014, is a novel approach of SBME [25]. RCDP is unique in that, it combines the most essential features of SBME: customized directive feedback and repetitive practice along with the principles of mastery learning. RCDP involves a migration in debriefing style, from the traditional post-simulation debrief to within-simulation directive feedback in the form of coaching, where the scenario is paused, learners are interrupted in their management and the instructor gives brief corrective instruction before the scenario resumes and learners continue, but this time, the "right" way.

Hunt, et al. describe RCDP as having three main principles. First is the principle of repeating “the right way”. Giving learners multiple chances to “do it right” is based on the education theories of overlearning, automatization and creating muscle memory [25]. Second is the principle of expert feedback. Faculty provides specific evidence-based feedback or expert-derived solutions for errors encountered during the simulation. The instruction occurs in real-time and is directed for feedback. The third is the principle of psychological safety. Hunt compares the learning environment to coaching world-class athletes. Instead of fearing mistakes, residents welcome the opportunity for coaching and practice time with the goal of becoming experts at saving lives [25].

### Objectives

a) Provide a review of the current status of RCDP research (definitions of RCDP, implementation strategies, and outcome measures) b) Identify gaps in RCDP understanding to guide future research.

## Review

### Methods

We followed a systematic review approach [28]. We designed a protocol compliant with the preferred reporting Items for systematic review and meta-analysis protocols (PRISMA-P) 2015 checklist [29].

The literature search occured between July and August 2016. We searched Ovid medical literature analysis and retrieval system online (MEDLINE) In-Process and Non-Indexed Citations and Ovid MEDLINE (1946–August 2016), excerpta medica database (Embase) (1980–August 2016), psychological information database (PsychINFO) (2002 to July 2016), Google Scholar, Web of Science and Scopus. See appendix for the full search strategy.

Since this is a relatively novel topic, we hand searched references and conducted a variety of internet searches with attention to the ‘grey literature’ to assemble published and unpublished resources. We hand searched the website ‘Society for Simulation in Health Care’, its journal and its affiliated organizations. We searched the contents and archives of specific journals (Advances in Simulation, BMJ Simulation and Technology-Enhanced Learning, Clinical Simulation in Nursing, Internet Journal of Medical Simulation, Cureus, Medical Teacher, Medical Education and Teaching and Learning in Medicine) using the terms “deliberate practice” or “rapid cycle deliberate practice”. We also searched the conference proceedings of multiple simulation and education conferences, such as International Pediatric Simulation Society Symposia and Workshop (IPSSW), International Meeting for Simulation in Healthcare (IMSH), International Conference on Residency Education (ICRE) and Canadian Conference on Medical Education (CCME) (2011-2016) using the same search terms and variations thereof.

**Figure 1:**
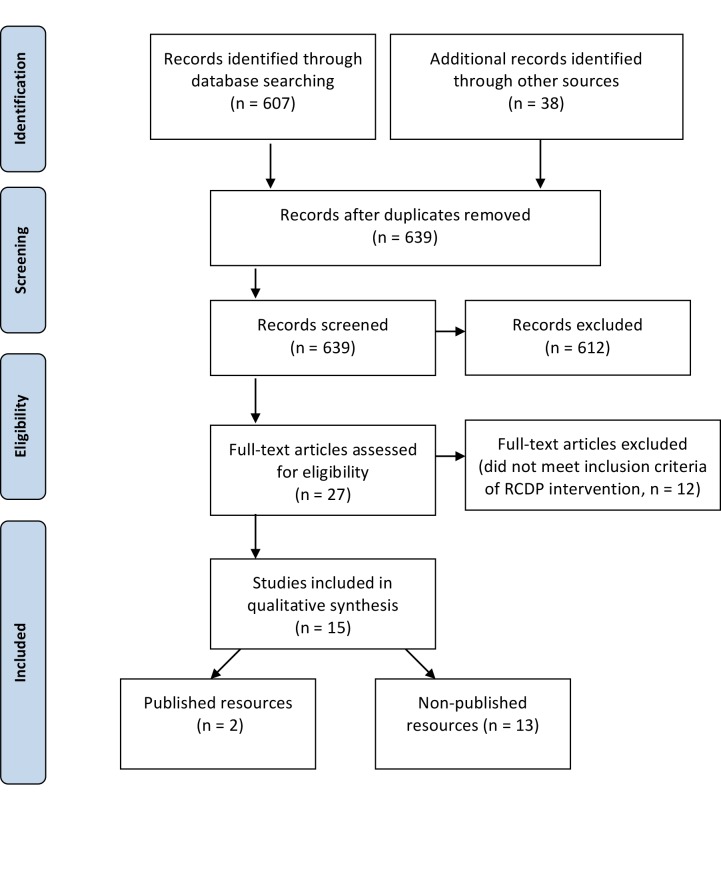
Flow diagram of study selection process

We independently screened the references by title and abstract. Common reasons to exclude articles were: focus other than health care education and rapid-cycle quality improvement reports. We obtained the full-text reports of all remaining trials and assessed them independently for eligibility, based on the defined inclusion criteria outlined in Table 1. Some full-text reports used deliberate practice to achieve mastery learning, however, lacked the other features of RCDP (i.e. microdebriefing or coaching style feedback or repetition or progressively challenging cases etc.) and consequently excluded the full resource selection process (Figure 1).

**Table 1:** Criteria for including studies

Inclusion criteria	
Participants	Learners in health care
Intervention	Rapid cycle deliberate practice (RCDP) healthcare simulation
Comparison	Traditional simulation, alternative instruction or no intervention
Outcomes	Impact on learner's reactions, knowledge, implementation in practice and patient outcome
Study design	Any trial design of any duration including non-indexed sources: abstracts, conference proceedings, instructor guides etc. English language publications

### Data synthesis

The identified materials were diverse, including qualitative and quantitative studies using both experimental and quasi-experimental methods, oral presentations, poster presentations and instructor guides for simulation education. Given the limited amount of randomized controlled trials and the diversity of the materials reviewed, we made no attempt to quantitate the results, grade the levels of evidence or perform statistical or meta-analysis. Instead, our focus was to examine the literature and provide a narrative review of RCDP. 

### Results of the search

**Figure 2:**
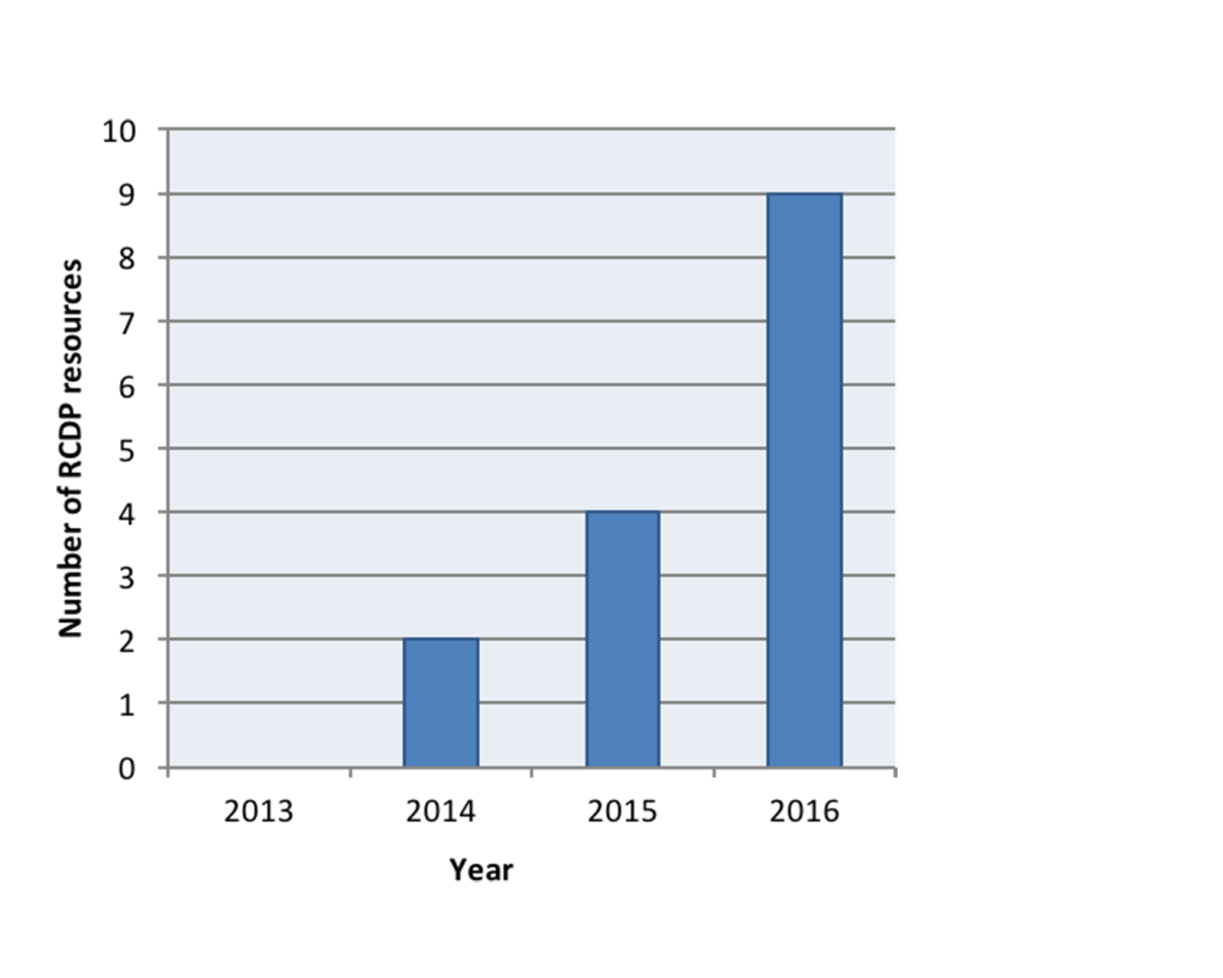
Rapid cycle deliberate practice scholarly output by year

RCDP is an emerging teaching method within the medical education community (Figure 2).

We identified 15 resources that met our inclusion criteria. The resources we identified are diverse and are summarized in Table 2. Resource characteristics are summarized in Table 3. The simulation skills that RCDP was used for are summarized in Table 4. 

 

**Table 2:** Types of resources identified

Type of resource	# of resources	Study ID
Published articles	2	[25, 35]
Poster presentations	6	[30-33, 36] (Lemke D, Fielder E, Hsu D, Doughty C. A pilot study of rapid cycle deliberate practice compared with traditional debriefing on interprofessional teams participating in the simulated acute care of infants. 14th Annual International Meeting on Simulation in Healthcare. San Francisco, CA. January 25-29th 2014)
Oral presentations	3	[34, 37] (Gross I, Noether J, Powell M, Bahar-Posey L: RCDP as a method to improve airway management skills in pediatric residents. International Network for Simulation-based Pediatric Innovation Research and Education (INSPIRE). 2016, Accessed: July 29, 2016: http://www.slideshare.net/INSPIRE_Network/new-alert-rapid-cycle-deliberate-practice-for-airway-management-in-pediatric-residents)
RCDP instructor guides	4	[38-41]

**Table 3:** Characteristics of identified resources Abbreviations: RCDP – Rapid cycle deliberate practice STAT – Simulation team assessment tool MCAF – Megacode checklist assessment form NRPE – Neonatal resuscitation performance evaluation DASH – Debriefing assessment for simulation in healthcare

Study Characteristic		# of studies	Study IDs
Study Design	Randomized control trial	3	[30-32]
	Pretest-posttest	5	[25, 33-36]
	Mixed	1	[37]
	Pilot	1	(Lemke, et al., 2014)
	Not yet defined	1	(Gross, et al., 2016)
	Instructor guide	4	[38-41]
Participants	Pediatric residents	5	[25, 30-31, 36] (Gross, et al., 2016)
	Pediatric emergency fellows	1	[34]
	Nurses	1	[35]
	Inter-professional teams	4	[32-33, 37] (Lemke, et al., 2014)
Intervention	RCDP	10	[25, 30-33, 35-37] (Lemke, et al. 2014) (Gross, et al., 2016)
	RCDP and traditional simulation	1	[34]
Comparison	Traditional simulation	7	[30-32, 36-37] (Lemke, et al. 2014) (Gross et al., 2016)
	Standard of Practice	1	[25]
	RCDP without "rewind"	1	(Gross, et al., 2016)
	None	3	[33-35]
Outcomes	Qualitative evaluations	4	[31, 33-35]
	STAT tool score	2	[30] (Lemke, et al., 2014)
	MCAF tool score	1	[31]
	NRPE tool score	1	[32]
	DASH tool	1	[34]
	Procedural skills checklists	2	[34] (Gross, et al., 2016)
	Procedure assessment (video)	1	(Gross, et al., 2016)
	Time to critical intervention	3	[25, 30, 32]
	Clinical reports	1	[35]

**Table 4:** Types of simulation skills employed by RCDP method

Type of simulation	# of studies	Study ID
Pediatric resuscitation	8	[25, 30, 34, 38-41] (Lemke, et al., 2014)
Pediatric resuscitation in resource-limited setting	1	[36]
Neonatal resuscitation	4	[31-33, 37]
First five minutes of cardiac arrest for nurses before code team arrives	1	[35]
Procedural skills (e.g. intubation, chest tube insertion, central line insertion)	2	[34] (Gross, et al., 2016)

### Definitions and descriptions of RCDP

**Figure 3:**
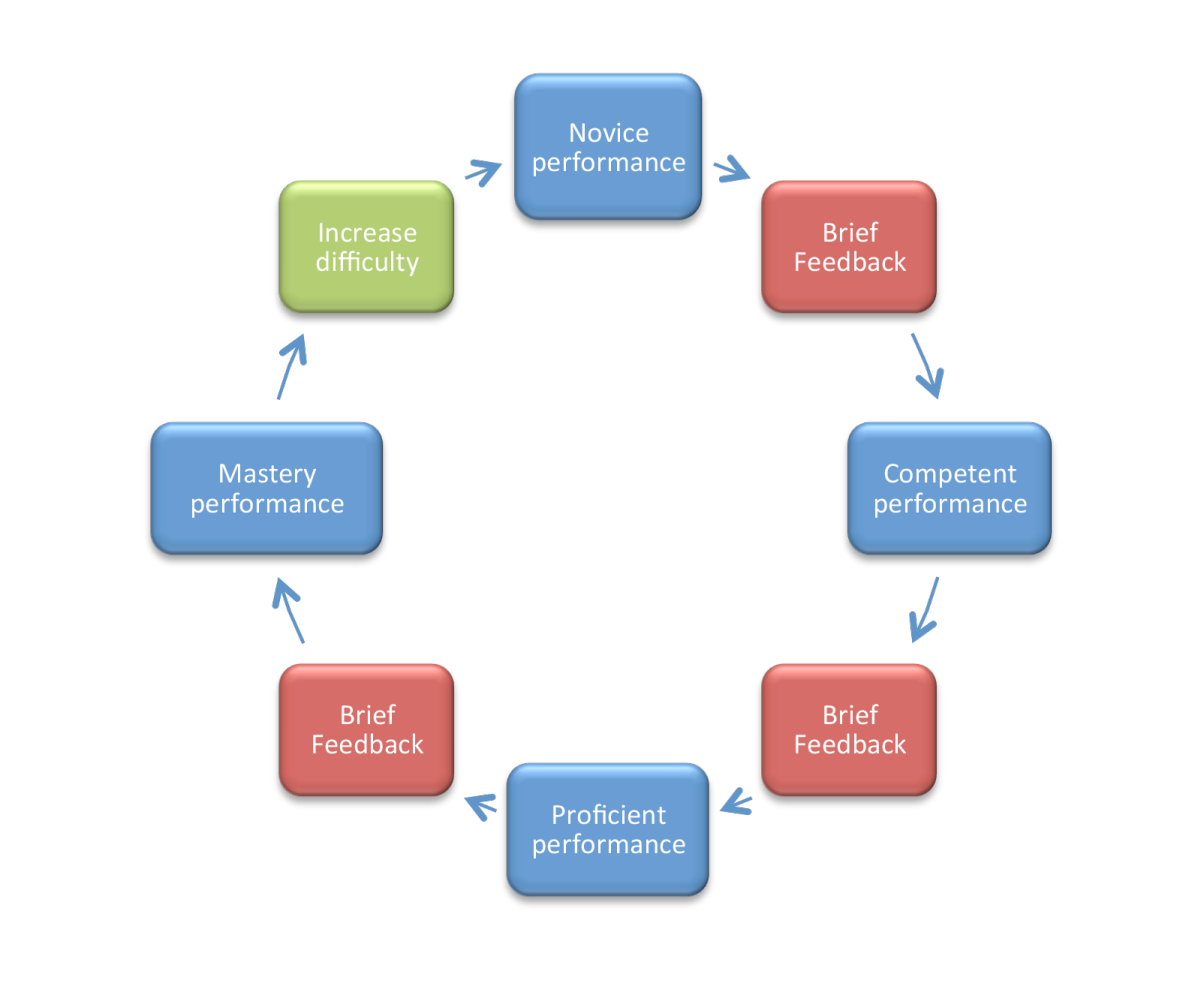
Rapid cycle deliberate practice model of learning After Lemke, et al. with permission (Lemke, et al., 2014)

Hunt, et al. describes RCDP as to “rapid cycle between deliberate practice and directed feedback until skill mastery is achieved” and then progress to more challenging scenarios [25]. All of the resources identified used a version of this definition when describing the RCDP teaching approach [25, 30-41]. Lemke, et al. depict the RCDP teaching model in Figure 3 (Lemke, et al., 2014).

### Implementation strategies and techniques of RCDP

Below, we outline techniques that are common to identify studies used during RCDP methodology. A) Splitting cases into small chunks of learnable skills: dividing a typical resuscitation case into smaller segments [25, 35, 38-40]; progress-limiting essential skill checklists [25, 35, 38-40]. 

B) Within-event debriefing or microdebriefing: a key component in the majority of resources were identified [25, 30-33, 35-37, 39-40]. Microdebriefing is a type of reflection-on-action that occurs within the simulation event [19]. A participant error precipitates a pause-correct (with rationale) rewind-replay cycle from the instructor [25]. Eppich, et al. outlined what microdebriefing would sound like based on Hunt’s example of an error during RCDP (Table 5) [19].

**Table 5:** Example of microdebriefing in rapid cycle deliberate practice After Eppich, et al. with permission [19]

Breached standard (pause before defibrillation <10 seconds)
“Okay guys, we just paused compressions for 15 seconds before the defibrillation and remember the AHA standard is no pause longer than 10 seconds and Dana Edelson’s paper [42] demonstrated that each five-second decrease in preshock pause is associated with a 86% increase in defibrillation success rate … so let me give you some strategies on how to shrink that pause and then we will rewind you and can try again.”

Techniques of within-event debriefing varied between studies. In Hunt’s study, the first scenario flowed uninterrupted without microdebriefing. Then instructors interrupted the scenarios for errors and addressed each error by identifying the breeched standard, providing solution oriented debriefing and scripted language to improve team communication and allowing participants to rewind 10 seconds and try again [25]. Kutzin’s microdebriefing included a task coaching session before rewinding and trying the scenario again [35]

**Figure 4:**
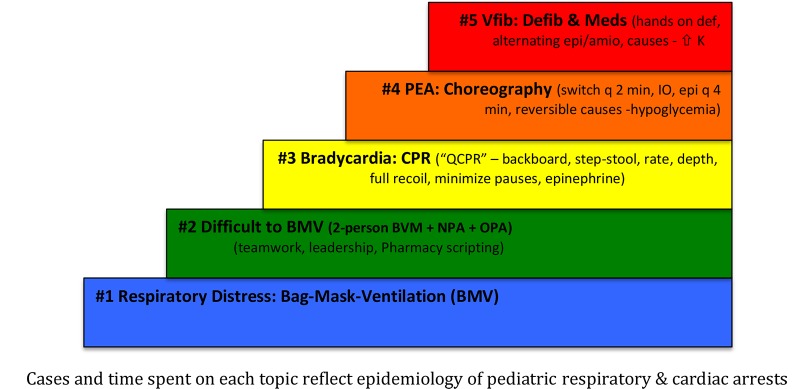
Clinical scenarios in progressive difficulty using Rapid Cycle Deliberate Practice - First Five Minutes (RCDP-FFM) After Hunt et al. with permission [25] BMV - bag mask ventilation NPA - nasopharyngeal airway OPA - oropharyngeal airway CPR - cardiopulmonary resuscitation QCPR - quality cardiopulmonary resuscitation PEA - pulseless electical activity IO - intraosseous access Epi - epinephrine Vfib - ventricular fibrillation Defib - defibrillation Meds - medications Def - defibrillator Amio - amiodarone K – potassium

C) Escalating difficulty: Hunt employed five clinical scenarios, each progressively more difficult and that built on previously mastered skills (Figure 4) [25].

Four additional studies identified in our search included multiple scenarios in the RCDP curriculum as opposed to a single RCDP session [32, 34, 36-37]. However, authors did not specify if these scenarios were progressively more challenging. 

### Outcome measures

Kirkpatrick describes four levels of impact from educational interventions (sometimes with subdivisions) [43]. These may be distilled to 1) learners’ perceptions or reactions to the activity 2) learning demonstrated objectively (e.g. in a test or the simulation laboratory) 3) real-life manifestation of learning (e.g. change in behaviour in the clinical environment) 4) results in the workplace (e.g. improved patient outcomes). These are termed K1 – K4 outcomes and are used below to categorize RCDP impact. We did not discover any evidence of RCDP having a K4 level impact.

*A) K1 Outcome Measures – Qualitative Evaluations: *Four studies used qualitative evaluations of participants’ attitudes towards RCDP as an outcome measure. Kutzin reported greater satisfaction and retention of the first five minutes of resuscitation after the RCDP education process [35]. Winter measured participants’ confidence and perceptions of teamwork using a six-point Likert scale pre- and post-RCDP training. Post-RCDP evaluations showed that learners had improved confidence in their role on the team, neonatal resuscitation program (NRP) algorithm knowledge, cardiopulmonary resuscitation (CPR) skills, Bag-valve-mask ventilation (BMV) skills and coordination of CPR/BMV ratio [33]. Sokol, et al. used immediate and delayed qualitative evaluations with Likert responses to measure participant perceptions of simulation styles and learning outcomes. Perceptions of post-event debriefing versus RCDP varied based on the level of participants experience and outcome being measured [37]. 

*B)* *K2 Outcome Measures – Established Scoring Tools: *Five studies used previously published tools to measure participant improvement before and after RCDP. Both Lemke and Welch-Horan used the simulation team assessment tool (STAT) to score residents’ performance. Arguments have been made for the validity of the STAT tool, as it was previously used to show a difference between novice and expert learners [44]. In Lemke’s pilot study on RCDP, the RCDP arm improved significantly compared to the traditional simulation arm in the team management subsection of the STAT tool (Lemke, et al., 2014). Welch-Horan’s primary outcome was team performance using the STAT tool after receiving either RCDP or traditional simulation and debriefing. They did not find significant differences in STAT scores between the two arms [30]. Other studies have used the megacode checklist assessment form (MCAF) [31], the neonatal resuscitation performance evaluation (NRPE) [32] and the debriefing assessment for simulation in healthcare (DASH) tool [34] although at the time of the current review, the results have not been published.

*C) K2 Outcome Measures – Procedural Assessment: *Two studies assessed participant’s procedural skill improvement after RCDP. Jeffers’ study, which combined RCDP and traditional debriefing, used the Chest Tube Insertion Competency Test (TUBE-iCOMPT), a new instrument to assess chest tube insertion skills. This study also used a procedural performance checklist for insertion of ultrasound guided internal jugular central line [34]. Gross’s study assessed intubation skills based on a procedural checklist used with videotaped intubations attempts (Gross, et al., 2016).

*D) K2 Outcome Measures – “Time-to” Active Skills: *Hunt’s prospective pre-test/post-test study used the time interval between onset of ventricular tachycardia and defibrillation as the primary outcome measure [25]. Rapid cycle deliberate practice first five minutes curriculum (RCDP-FFM) was associated with a decrease in no-flow fraction and no-blow fraction. After RCDP-FFM, residents were 1.7 times more likely to defibrillate within two minutes per American Heart Association (AHA) guidelines. As well, there was a 10-fold reduction in the median pre-shock pause [25]. Welch-Horan’s randomized control trial measured time to CPR, time to defibrillate or time to first epinephrine dose as secondary outcomes. This study showed no statistically significant differences between RCDP and traditional simulation groups in times of critical interventions [30]. Patricia’s cluster randomized control trial measures timing of active skills such as time to intubation, time to chest compression and time to umbilical vein catheter (UVC) placement in a post-training simulation immediately after learners receive either RCDP or traditional simulation training [32].

*E) K3 Outcome Measures – Clinical Reports: *One study used clinical reports as an outcome measure for assessing RCDP. Kutzin reported that nurses self-reported as better prepared to manage real patients in cardiac arrest after RCDP training [35].

### Strengths and limitations of this review

A strength of this article is that, it is the first to summarize and evaluate existing literature (published and non-published) on RCDP, which will help medical educators understand RCDP as a teaching method as well as help guide future research on RCDP. We were rigorous in our avoidance of publication bias and searched the grey literature extensively.

Given its prominence in the consciousness and conversation of educators, we expected to find more literature on the topic of RCDP. A limitation of the review is the quantity and quality of the material summarized, indicative of the infancy of the discipline. The rapid emergence of material for the last three years leads us to suspect that a similar review conducted two years from now would yield significantly more material.

## Conclusions

RCDP is a novel teaching approach in simulation-based medical education. We are just beginning to understand its efficacy, appropriate indications, and how it compares to other types of simulation-based learning. The education community is consistent in its definition of RCDP but varies in terms of manifestation and impact.

The central tenets are providing learners with multiple opportunities to practice the right way and using directive feedback (microdebriefing) within the scenario. Chunking scenarios and escalating difficulty are common implementation techniques. Various outcome measures were used by the identified studies, such as qualitative assessments, scoring tools, procedural assessments, time to active skills and clinical reports and the results were inconsistent. 

Further research should focus on retention in RCDP, translation into clinical behaviors, impact on patient care and whether it is superior to traditional SBME in these regards. Furthermore, future research should diversify the scenarios from pediatric and neonatal resuscitation skills to include adult resuscitation scenarios and broaden the participant population from trainee physicians and nurses to include other licensed practitioners from a range of disciplines and specialties.

## Appendices

Search Strategy

Database: Ovid MEDLINE(R) In-Process & Other Non-Indexed Citations and Ovid MEDLINE(R) <1946 to Present>

Search Strategy:

--------------------------------------------------------------------------------

1 RCDP.ti,ab. (93)

2 "deliberate practice".ti,ab,kf,kw,tw. (330)

3 ("rapid cycle" adj3 (feedback or feed back or practice)).ti,ab,kf,kw. (13)

4 1 or 2 or 3 (432)

5 limit 4 to (english language and yr="2006 -Current") (332)

6 remove duplicates from 5 (323)

7 music.mp. or musical.ti. or sport*.ti (35895)

8 6 NOT 7 (305)

***************************

Database: Embase <1980 to 2016 Week 33>

Search Strategy:

--------------------------------------------------------------------------------

1 RCDP.ti,ab. (113)

2 "deliberate practice".ti,ab,kw,tw. (423)

3 ("rapid cycle" adj3 (feedback or feed back or practice)).ti,ab,kw. (25)

4 1 or 2 or 3 (557)

5 limit 4 to (english language and yr="2006 -Current") (451)

6 *music/ (7989)

7 music:.ti. (10513)

8 *sport/ (20357)

9 sport.ti. (7217)

10 6 or 7 or 8 or 9 (37665)

11 5 not 10 (439)

12 remove duplicates from 11 (420)

***************************

Database: PsycINFO <2002 to July Week 4 2016>

Search Strategy:

--------------------------------------------------------------------------------

1 RCDP.ti,ab. (0)

2 "deliberate practice".ti,ab,kf,kw,tw. (312)

3 ("rapid cycle" adj3 (feedback or feed back or practice)).ti,ab,kf,kw. (2)

4 1 or 2 or 3 (314)

5 limit 4 to (english language and yr="2006 -Current") (259)

6 remove duplicates from 5 (259)

7 music.mp. or musical.ti. or sport*.ti. [mp=title, abstract, heading word, table of contents, key concepts, original title, tests & measures] (25090)

8 6 not 7 (214)

***************************
